# The Multiple Neurovascular Pathologies in Older Adults Living with and without Peripheral Insulin Resistance

**DOI:** 10.1002/alz.089870

**Published:** 2025-01-09

**Authors:** Rupal I. Mehta, Ana W. Capuano, Roshni Biswas, David A. Bennett, Zoe Arvanitakis

**Affiliations:** ^1^ Rush Alzheimer’s Disease Center, Rush University Medical Center, Chicago, IL USA; ^2^ Rush Alzheimer’s Disease Center, Chicago, IL USA

## Abstract

**Background:**

The overall odds and permutations of neurovascular pathologies (NVP) in older adults living with diabetes or peripheral insulin resistance (PIR) have not been comprehensively investigated. Moreover, specific biological mechanisms linking apolipoprotein E (ApoE) genotype, NVP, and Alzheimer’s disease (AD) remain unclear. Here, we examine the odds and permutations of NVP in older adults with PIR in relation to those without.

**Materials and Method:**

This study evaluated older adults who were followed in longitudinal studies of aging and underwent autopsy. Annual clinical evaluations included data to classify PIR status by history, direct medication inspection, and/or blood hemoglobin A1C level (≥6.5%). Neuropathological examinations were systematically performed upon death and included evaluation for NVP and AD pathology. Logistic regression models were used to analyze the association of PIR with the presence of moderate to severe NVP as well as NVP permutations, respectively, in persons with or without ApoE ε2 or ε4 allele and/or AD.

**Result:**

A total of 1997 older adults (404 with PIR) were studied. Participants died at mean age of 89 years (SD = 6.81 years). Overall, persons with PIR had higher odds of chronic macroinfarcts (odds ratio (OR), 1.33 [95% CI, 1.08–1.65]) and chronic microinfarcts (OR, 1.37 [95% CI, 1.10‐1.71]) but exhibited lower odds of CAA (OR, 0.74 [95% CI, 0.59–0.92]). Analysis of NVP permutations revealed that single pathologies (with absence of others) were most common, followed by combined microinfarcts plus macroinfarcts. Persons with PIR had higher odds of microinfarcts alone (OR, 1.56 [95% CI, 1.02–2.37]), macroinfarcts alone (OR, 1.54 [95% CI, 1.01‐2.35]), and combined microinfarcts with macroinfarcts (OR, 2.02 [95% CI, 1.21‐3.35]). However, the odds of microinfarcts were not significantly higher in persons with PIR when controlling for the presence of ApoE ε2 allele, and only the presence of both types of infarcts together was significantly higher in PIR after controlling for the presence of ApoE ε4 allele with or without AD.

**Conclusion:**

This study examined multiple NVP in persons with and without PIR. It shows that older adults living with PIR, relative to those without, have higher odds of microinfarcts and macroinfarcts in the absence of CAA, atherosclerosis and arteriolosclerosis.

